# Quantization Effects on Complex Networks

**DOI:** 10.1038/srep26733

**Published:** 2016-05-26

**Authors:** Ying Wang, Lin Wang, Wen Yang, Xiaofan Wang

**Affiliations:** 1 Department of Automation, Shanghai Jiao Tong University, and Key Laboratory of System Control and Information Processing, Ministry of Education of China, Shanghai 200240, P. R. China; 2 Key Laboratory of Advanced Control and Optimization for Chemical Processes (East China University of Science and Technology), Ministry of Education, Shanghai 200237, P. R. China

## Abstract

Weights of edges in many complex networks we constructed are quantized values of the real weights. To what extent does the quantization affect the properties of a network? In this work, quantization effects on network properties are investigated based on the spectrum of the corresponding Laplacian. In contrast to the intuition that larger quantization level always implies a better approximation of the quantized network to the original one, we find a ubiquitous periodic jumping phenomenon with peak-value decreasing in a power-law relationship in all the real-world weighted networks that we investigated. We supply theoretical analysis on the critical quantization level and the power laws.

In essence, most real-world networks are weighted networks in the sense that different edges may actually have different weights^1^,^2^,^3^. However, many networks we constructed and investigated in network science studies are based on quantized weights of edges for convenience or due to limitation on measurement. For example, although different ties in a social network may have different strengths, one usually views a social network as an unweighted network in which there is an edge of unit weight between a pair of people if and only if they are friends^4^. Sometimes one uses multiple different weights to represent strong or weak ties in a social network based on, for example, the number of communications between a pair of people^5^. A natural question is: what are the differences between the original network and its corresponding quantized network? Or, to put it in another way, how may the quantization affect the properties and behaviors of a network?

Quantization analysis has long been an important problem in modern science and technology^6^. In this work, our major goal is to show the quantization effects on the spectrum of the Laplacian of a network, since the Laplacian determines many dynamical behaviors of a network such as consensus^7^,^8^,^9^ and synchronization^10^,^11^. Periodic jumping of eigenvalues with peak-value decreasing in a certain power-law relationship is found in many real-world weighted networks. Theoretical estimations of the critical quantization level and the peak values are obtained. It is found, from quantization effects on the degree-preserving and fully randomized networks, that the critical quantization level is determined by the degree distribution.

## Quantization Procedure

Consider an undirected weighted network *G* = (*V*, *E*, *W*) with |*V*| = *N* nodes and |*E*| = *M* edges, where *W* = (*w*_*ij*_)_*N*×*N*_ is a nonnegative weighted adjacency matrix: *w*_*ij*_ > 0 if and only if there is an edge between nodes *i* and *j*.

In order to facilitate the comparisons among different networks, the edge weights are firstly normalized to 
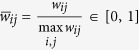
, and the normalized network is denoted as 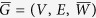.

**Figure 1:**
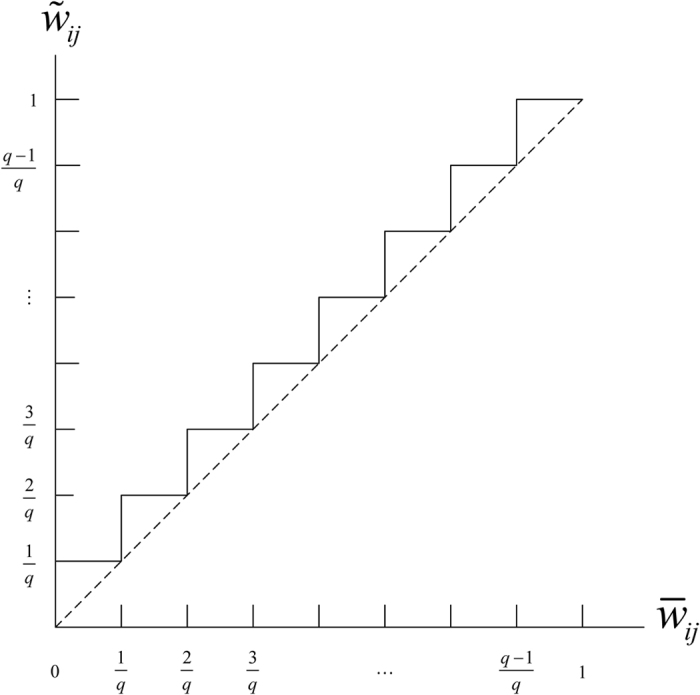
Schematic of the quantization law.

Then, divide the interval (0, 1] into *q* evenly-spaced intervals, as 
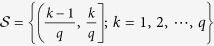
, where *q* ∈ N_+_ represents the quantization level. And, each nonzero normalized weight 
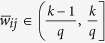
, for *k* = 1, 2, ···, *q*, is quantized as 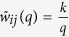 (Fig. 1). The resulting quantized network is denoted as 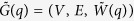.

If *q* = 1, the original weighted network reduces to a network with edges of unit weight, in which 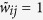 if there is an edge between nodes *i* and *j*; otherwise, 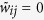. Intuitively, a larger quantization level *q* should imply a better approximation of the quantized network to the original one. However, it will be shown below that this intuition is not entirely correct. There is a series of critical levels at which the best quantization result can be obtained.

Consider a connected network *G* (for a disconnected network, we may consider its largest connected component). The eigenvalues of its Laplacian *L* can be ordered as 0 = *λ*_1_ < *λ*_2_ ≤··· ≤ *λ*_*N*_, where *L* = *S* − *W* and *S* = diag(*s*_1_, *s*_2_, ···, *s*_*N*_) is a diagonal matrix with strengths 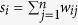. The second smallest eigenvalue *λ*_2_ is called the algebraic connectivity of the network *G*, which also characterizes the convergence speed of consensus algorithms in the sense that a larger *λ*_2_ implies a faster speed^12^. The eigenratio 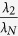 is frequently used to characterize the synchronizability of a network in the sense that a larger eigenratio implies a stronger synchronizability^13^,^14^.

The eigenvalues of the Laplacian 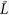 of the normalized network 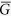 are denoted as 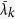, while the eigenvalues of the quantized network 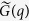 with the quantization level *q* are denoted as 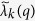, *k* = 1, 2, ···, *N*.

## Main Results

### Jumping phenomenon

**Figure 2:**
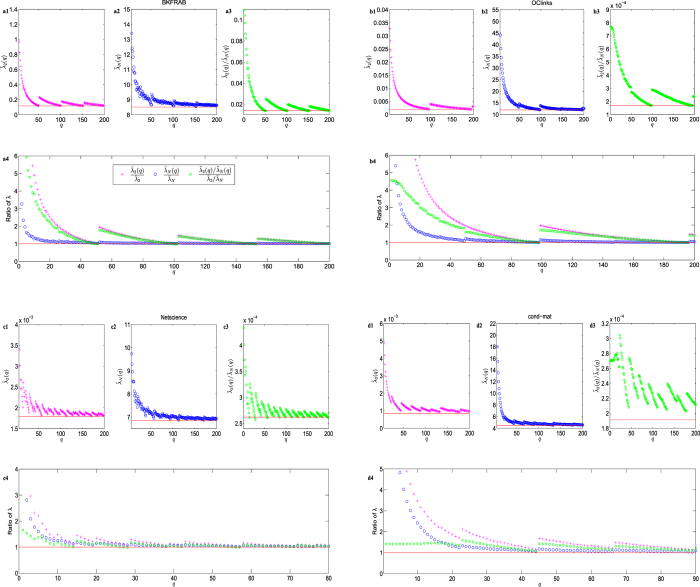
Quantization on real-world networks. (**a**) The BKFRAB network. (**b**) The OClinks network. (**c**) The Netscience network. (**d**) The cond-mat network. The behaviors of eigenvalues 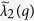, 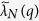 and 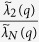 are plotted by the symbol plus, circle and hexagon in (**a–d**) (1–3), respectively. 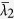, 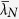 and 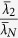 of 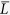 are also drawn as the red reference line in (**a–d**) (1–3), respectively. (**a–d**) (4) displays the ratio of 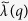 and 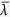, where the red reference line has value 1.

Figure 2 and Supplementary Fig. S1 reveal a periodic jumping phenomenon in the quantization effects on the investigated real networks. In each case, there exists a corresponding critical quantization level q^*^ such that, as the quantization level *q* increases from 1 to q^*^, both 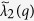 and 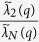 decrease monotonically toward 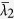 and 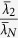, respectively. However, for *q* = q^*^ + 1, both 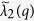 and 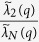 jump to some peak values and then decrease monotonically again till *q* = 2q^*^, and so on. Such a jumping phenomenon repeats periodically with period q^*^, which is found in all real-world networks investigated.

The quantization procedure is implemented on nine representative real-world networks drawn from diverse fields, including one interaction network (BKFRAB), one coappearance network (Lesmis), one social networks (OClinks), five scientific collaboration networks in different disciplines (Geom, astro-ph, hep-th, Netscience and cond-mat), and one news network (DaysAll). Details of the networks are described in Methods as Properties of real-world networks. Their topological features such as degree distribution, weighted degree distribution and edge weight distribution are shown in Supplementary Figs S5–S7. Table 1 shows the general information of these nine real-world networks. The first five networks have all positive integer weights, and the remaining four networks have some non-integer weights. The experimentally observed real critical quantization level is denoted by 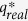. For each real network, we compute the critical quantization level 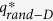 of its corresponding degree-preserving randomized network (see Methods about Degree-preserving randomization and Supplementary Fig. S2) and 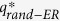 of its corresponding fully randomized network (see Methods about Full randomization and Supplementary Fig. S3). Both of the randomizations did not change the edge weight distribution and the jumping phenomenon. In all the cases, 
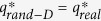
, but 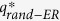 does not always equal to 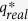, which means that the critical quantization level is determined by the degree distribution.

### Critical quantization level

Since 
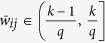
, one has 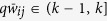, and the quantized weight can thus be expressed as 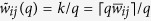, where 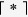 represents the smallest integer larger than or equal to *. Denote 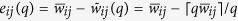. Clearly, if 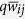 is an integer, then *e*_*ij*_(*q*) = 0; otherwise, 
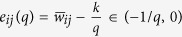
.

**Figure 3:**
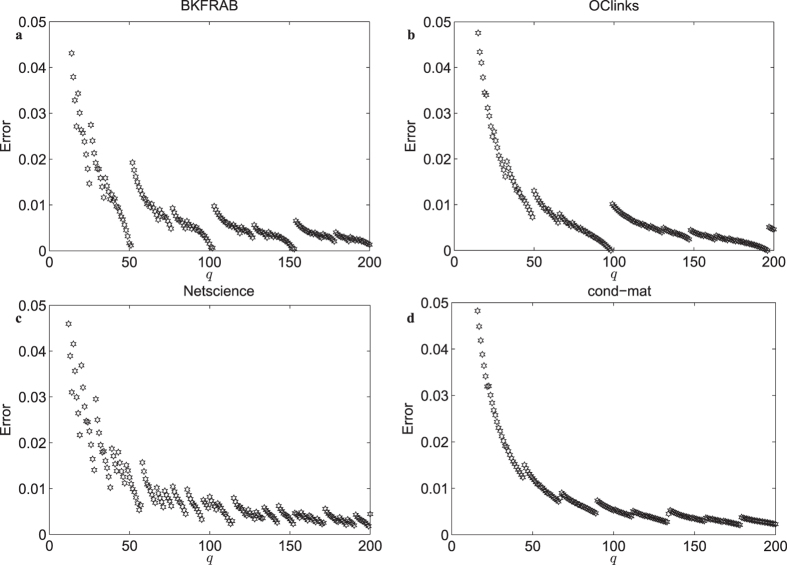
Behavior of the normalized *E*_*L*_. (**a**) The BKFRAB network. (**b**) The OClinks network. (**c**) The Netscience network. (**d**) The cond-mat network.

Define the difference between the Laplacian of the normalized and the quantized network as 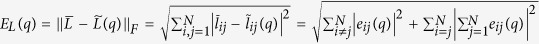, where 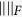 is the Frobenius norm, so *E*_*L*_(*q*) = 0 if and only if *e*_*ij*_(*q*) = 0 for all *i* and *j*. Figures 3(a–d) depict the behavior of the normalized *E*_*L*_ (divided by the maximum value), demonstrating that the jumping phenomenon of the eigenvalues could be attributed to the jumping phenomenon of *E*_*L*_. As *q* increases from 1 to q^*^, *E*_*L*_ decreases monotonically. However, for *q* = q^*^ + 1, *E*_*L*_ jumps to some peak values and then decreases monotonically again till *q* = 2q^*^, and so on.

Now, consider a network with all integer weights. Theoretically, one could speculate that


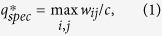


where *c* is the greatest common divisor of all *w*_*ij*_. Such a critical quantization level will lead to 
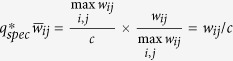
, which is an integer, and thus *e*_*ij*_(*q*) = 0 as well as *E*_*L*_(*q*) = 0, which implies no differences between the Laplacians of the normalized and of the quantized network. Specially, if all *w*_*ij*_ are coprime without any common divisors among them, then *c* = 1.

### Power-law decreasing of the peak values

As observed in the jumping phenomenon, 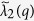 and 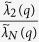 will jump to some peak values from zq^*^ to zq^*^ + 1 (*z* ∈ *N*_+_) with period q^*^, where the peak values are calculated according to 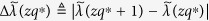.

**Figure 4:**
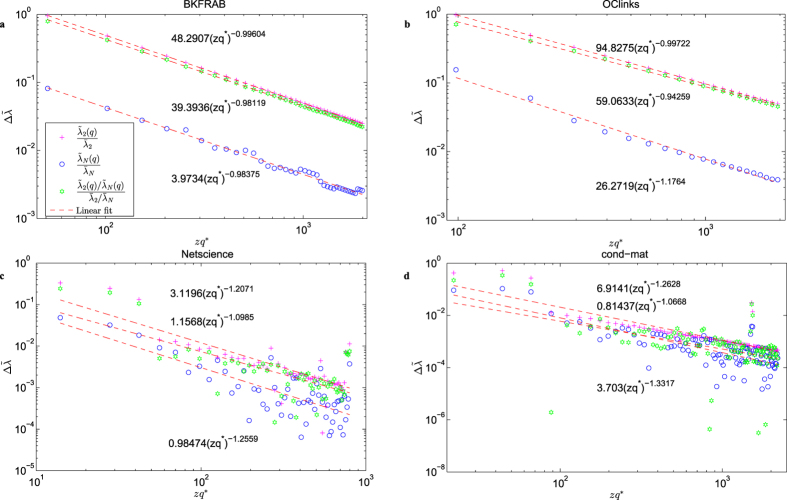
Peak values 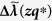 v.s. zq^*^. (**a**) The BKFRAB network. (**b**) The OClinks network. (**c**) The Netscience network. (**d**) The cond-mat network. Each curve is almost linear in the double logarithmic coordinates with nearly the same slope. The red linear fit line is also given with fitting parameters listed aside.

Figure 4 and Supplementary Fig. S4 show how the peak value 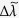 decreases with zq^*^ in a power-law relationship. Each curve is almost linear in the double logarithmic coordinates, with nearly the same slope, i.e., 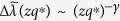, with *γ* ≈ 1. The linear fit (red-dashed line) is depicted in the figure and the corresponding power exponents are shown in Table 2.

Such relationship between 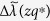 and zq^*^ (Table 2) suggests that, as the quantization level increases, the peak values decrease faster and finally converges to zero asymptotically, which helps to predict the variation trend of the peak values and further to estimate the value of 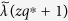.

### Estimation of the peak values

The perturbation approach^15^ is applied to estimate the peak values 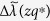. Suppose that the Laplacian 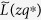 is perturbed by 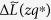 to 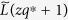, i.e., 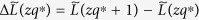. Then, the variation of the *k*-th eigenvalue of 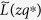 after the perturbation is Δ*λ*_*k*_. Note that 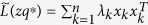, where *λ*_*k*_ and *x*_*k*_ are the eigenvalue and the corresponding orthogonal normalized eigenvector, respectively. If 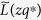 is non-degenerate with distinct eigenvalues, the perturbation of eigenvalues could be estimated according to


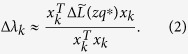


Next, select twenty zq^*^ (*z* = 1, 2, …, 20) for each network to evaluate the accuracy of the estimation. Define the real peak values (with the symbol ~) and the estimated peak values (with the symbol ^) of eigenvalues as follows:


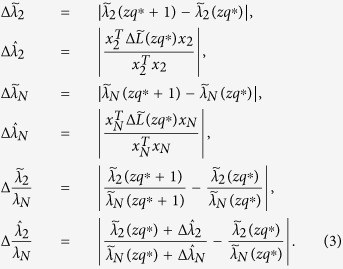


Statistics in Table 3 demonstrate the effectiveness of the method on estimating peak values of eigenvalues in the investigated real networks, which implies that one can foreknow the behaviors of the quantized networks as *q* increases from zq^*^ to zq^*^ + 1.

### Explanation of the relationship between △ 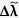 and *zq* ^*^

The above estimation method gives a hint to explain the relationship between 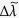 and zq^*^. Since 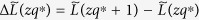, and each element 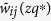 of 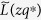 is 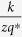, *k* = 1, 2, ···, zq^*^, it is possible to determine the element 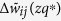 of 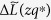.

**Figure 5:**
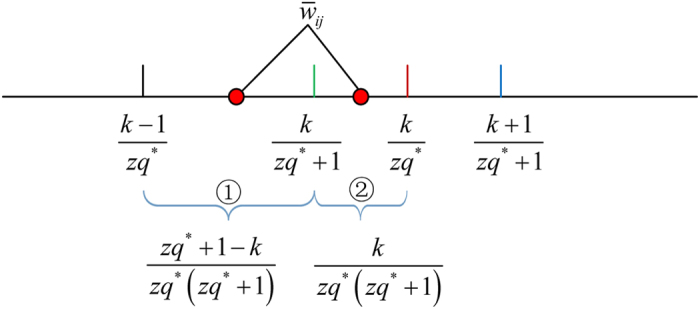
Illustration of the quantization of 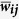 in 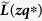 and 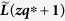. The red circles represent two possible positions of 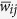 in segments ① ②, respectively, which therefore correspond to two different quantized values 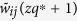 in 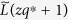.

As Fig. 5 shows, given one normalized weight 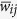 to be quantized, there are only two cases to consider. (Case I): if 
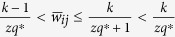
 (segment ①), it will be quantized to 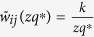 in 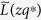, but 
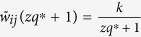
 in 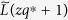. In this case, an element-wise error between the two matrices is 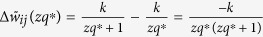.

(Case II): If 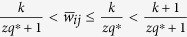 (segment ②), it will still be quantized to 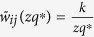 in 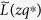, but 
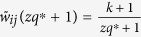
 in 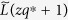, which leads to an element-wise error between the two matrices as 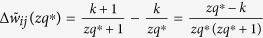.

By analyzing the complementary cumulative distribution of the normalized edge weights in the networks, one finds that it obey the power-law distribution (Supplementary Fig. S5). This implies that most 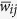 are very small such that the value of *k* in the quantized weight 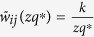 will be small. Ignoring the few 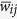 with large values close to 1, it is reasonable to assume that *k* ≪ zq^*^. Hence, as zq^*^ → ∞, one has 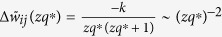 (Case I) and 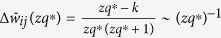 (Case II).

Furthermore, by comparing the segments in Fig. 5, the length of segment ① will be longer than ② as zq^*^ → ∞, which implies a bigger chance in Case I. Therefore, all weights in 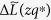 should have a distribution as 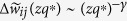, where *γ* ∈ (1, 2). Then, one could derive 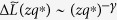, and 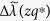 is approximately proportion to (zq^*^)^−*γ*^ according to equation (2).

### Influence of the normalization

If we do not normalize the edge weights firstly, the quantization procedure will be as follows: Divide the interval 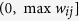 into *q* evenly-spaced intervals, as 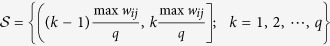, where *q* ∈ N_+_ represents the quantization level and max *w*_*ij*_ denotes the maximum edge weight, respectively. Then, each nonzero edge weight 
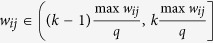
, for *k* = 1, 2, ···, *q*, is quantized as 
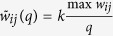
. Supplementary Figs S8 and S9 display the jumping and power-law phenomena in the original networks without the normalization process, which reveal that such phenomena are caused by quantization rather than normalization. In summary, for each original weighted network and its corresponding normalized version, we have the following findings: i) They both display jumping phenomenon with the same critical quantization level q^*^; ii) They both display power-law phenomenon with the same power exponent *γ*; iii) The difference just lies in that they have different spectrum.

## Conclusion

Quantization is generally utilized in constructing and investigating weighted networks in practice. In this work, we investigate how network behaviors are affected by quantization. Intuitively, larger quantization levels would preserve more information. However, our experiments on real-world weighted networks exhibit a periodic jumping phenomenon and peak-value decreasing in a certain power-law relationship. We therefore find that the critical quantization level leads to a better approximation of the quantized network to the original one. We also give theoretical analysis on the jumping phenomenon and the power-law property. However, we still lack theoretical analysis of the critical quantization level for networks with non-integer weights. Moreover, we infer that the jumping phenomenon is essentially related to the edge weight distribution of a network, which deserves a further investigation. It should also be noted that we only investigate the influence of quantization on the eigenvalues of the Laplacian of a network, so more works are needed to characterize the influence of quantization on other properties and behaviors of a network in future studies.

## Methods

### Degree-preserving randomization

The randomization procedure^16^ keeps the degree of every node unchanged (rand-Degree). Suppose that *w*_*ij*_ denotes the weight on the edge between node *v*_*i*_ and *v*_*j*_. Given two random non-overlapping edges between *v*_1_*v*_2_ (weight *w*_12_) and *v*_3_*v*_4_ (weight *w*_34_), whose endpoints do not intersect, the degree-preserving randomization will either result in two new edges *v*_1_*v*_3_ with weight *w*_12_ and *v*_2_*v*_4_ with weight *w*_34_, or two new edges *v*_1_*v*_4_ with weight *w*_12_ and *v*_2_*v*_3_ with weight *w*_34_.

### Full randomization

Full randomization procedure turns a network into an Erdös-Rényi random network (rand-ER)^17^,^18^, with the number of nodes (*N*) and the number of edges (*M*) unchanged. Randomly select one existing edge *v*_1_*v*_2_ and two nodes *v*_3_ and *v*_4_, whose endpoints do not intersect. If no edge between *v*_3_*v*_4_ exists, it is newly created and be assigned with weight *w*_12_, meanwhile edge *v*_1_*v*_2_ is deleted.

### Properties of real-world networks

**BKFRAB** is a human interactions network among students living in a fraternity at a West Virginia college. It records the number of times a pair of subjects (residents in the fraternity from three months to three years) were seen in conversation by an observer.

**Lesmis** is a weighted network of coappearances of characters in Victor Hugo’s novel “Les Miserables”. Nodes represent characters and edges connect a pair of characters that appear in the same chapter of the book. The values on the edges are the number of such coappearances.

**Geom** is a collaboration network in computational geometry. Two authors are linked with an edge, if and only if they had a joint publication. The weight of an edge is the number of joint works.

**OClinks** is an online weighted social network. It is created from an online community at the University of California, Irvine, which covers the period from April to October 2004. Nodes represent students, and edges are the online messages between them. The weight of an edge is defined as the number of messages sent.

**DaysAll** is a Reuters terror news network, which is based on all stories released during 66 consecutive days by the news agency Reuters concerning the September 11 attack on the U.S. The nodes of the network are words (terms); there is an edge between two words if and only if they appear in the same text unit (sentence). The weight of an edge is its appearance frequency.

**astro-ph** is a weighted network of coauthorships between scientists posting preprints on the Astrophysics E-Print Archive between January 1, 1995 and December 31, 1999. The network is weighted, with weights assigned as described in the original papers. The network used here is just the largest component.

**hep-th** is a weighted network of coauthorships between scientists posting preprints on the High-Energy Theory E-Print Archive between January 1, 1995 and December 31, 1999. The network is weighted, with weights assigned as described in the original papers.

**Netscience** is a coauthorship network of scientists working on network theory and experiments. The network was compiled from the bibliographies of two review articles on network science, with a few additional references added by hand. The version used here is just the largest component of 379 scientists but not all components of the network with a total of 1589 scientists because the latter is not a connected graph.

**cond-mat** is a weighted network of coauthorships between scientists posting preprints on the Condensed Matter E-Print Archive between January 1, 1995 and December 31, 1999. The network is weighted, with weights assigned as described in the original papers. The network used here is just the largest component.

## Additional Information

**How to cite this article**: Wang, Y. *et al.* Quantization Effects on Complex Networks. *Sci. Rep.* **6**, 26733; doi: 10.1038/srep26733 (2016).

## Supplementary Material

Supplementary Information

## Figures and Tables

**Table 1:** Quantization on real-world networks.

Network	*N*	*M*	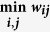	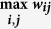	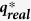	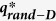	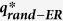
BKFRAB^19^	58	967	1	51	51	51	31 ± 14
Lesmis^20^	77	254	1	31	31	31	25 ± 8
Geom^21^	3621	9461	1	77	77	77	70 ± 16
OClinks^22^	1893	13835	1	98	98	98	98 ± 0
DaysAll^23^	13308	148035	1	745	745	745	745 ± 0
astro-ph^24^	14845	119652	0.0179	16.5	66	66	46 ± 26
hep-th^24^	5835	13815	0.0556	34.0111	102	102	68 ± 32
Netscience^25^	379	914	0.1250	4.75	14	14	15 ± 7
cond-mat^24^	13861	44619	0.0588	22.3333	22	22	22 ± 0

For each network, the listed properties include: names and references; number of nodes (*N*) and edges (*M*); minimum and maximum edge weights 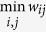, 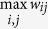; critical quantization level 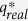 of the real networks, 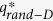 of the degree-preserving randomized networks; critical quantization level 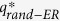 of the fully randomized networks, denoted as *μ* ± *σ*, where *μ* is the mean value and *σ* is the standard deviation of ten realizations.

**Table 2:** Power exponents *γ* of the peak values.

Network	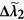	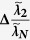	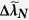
BKFRAB	0.99604	0.98119	0.98375
Lesmis	0.9713	0.94116	0.998
Geom	0.96135	0.96522	0.99142
OClinks	0.99722	0.94259	1.1764
DaysAll	0.99965	0.96628	0.9972
astro-ph	1.0188	1.0047	0.8650
hep-th	1.0355	1.0134	0.99576
Netscience	1.2071	1.0985	1.2559
cond-mat	1.2628	1.0668	1.3317

**Table 3:** Estimation error.

Network	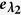	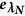	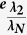
BKFRAB	0.0000 ± 0.0001	−0.0003 ± 0.0008	0.0000 ± 0.0000
Lesmis	0.0002 ± 0.0004	−0.0008 ± 0.0023	0.0000 ± 0.0001
Geom	0.0000 ± 0.0000	−0.0001 ± 0.0003	0.0000 ± 0.0000
OClinks	0.0000 ± 0.0000	−0.0607 ± 0.2127	0.0000 ± 0.0000
DaysAll	0.0000 ± 0.0000	−0.0003 ± 0.0007	0.0000 ± 0.0000
astro-ph	0.0000 ± 0.0000	−0.0001 ± 0.0002	0.0000 ± 0.0000
hep-th	0.0000 ± 0.0000	−0.0000 ± 0.0000	0.0000 ± 0.0000
Netscience	0.0000 ± 0.0001	−0.0002 ± 0.0008	0.0000 ± 0.0000
cond-mat	0.0000 ± 0.0001	−0.0086 ± 0.0376	0.0000 ± 0.0000

The errors between the real and the estimated eigenvalues are defined as 
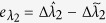
, 
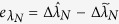
, and 
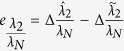
. The mean value *μ* and standard deviation *σ* of the three kinds of errors are listed as *μ* ± *σ*.
