# Supplementary material for: Quantization Effects on Complex Networks
Source: Sci Rep. 2016 May 26;6:26733. doi: 10.1038/srep26733 (PMC4881046; doi:10.1038/srep26733)
Supplement: Supplementary Information [file srep26733-s1.pdf]

Quantization Effects on Complex Networks

Supplementary Information

Ying Wang[1], Lin Wang[1], Wen Yang[2] & Xiaofan Wang[1*]

[1]Department of Automation, Shanghai Jiao Tong University, and Key Laboratory of System Control and Information Processing, Ministry of Education of China, Shanghai 200240, P. R. China, [2]Key Laboratory of Advanced Control and Optimization for Chemical Processes (East China University of Science and Technology), Ministry of Education, Shanghai 200237, P. R. China

SUPPLEMENTARY FIGURE

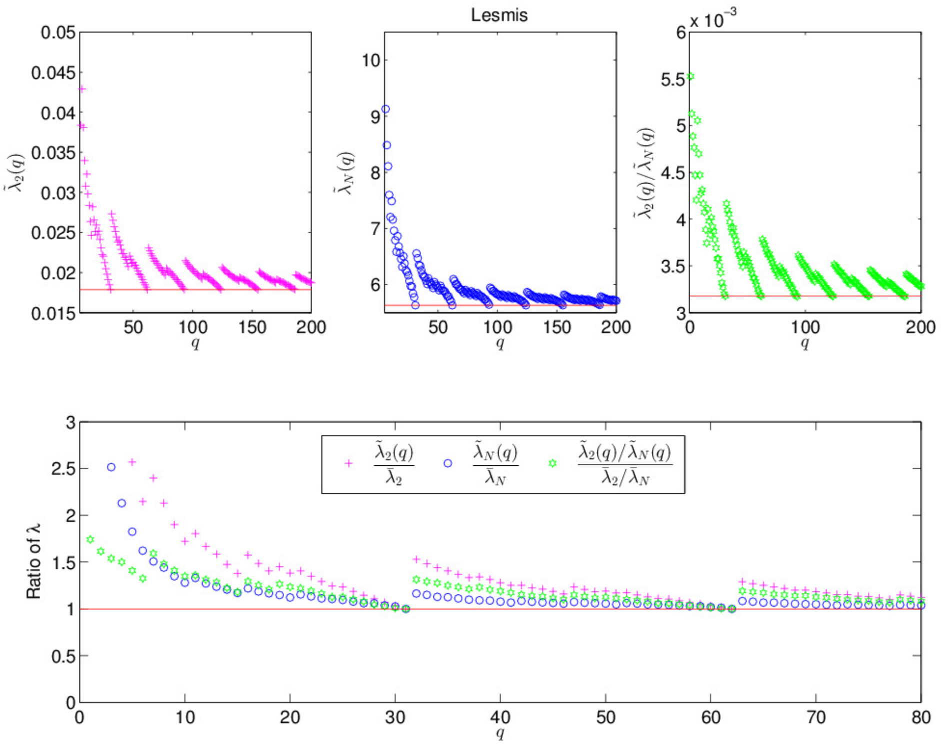

(a)

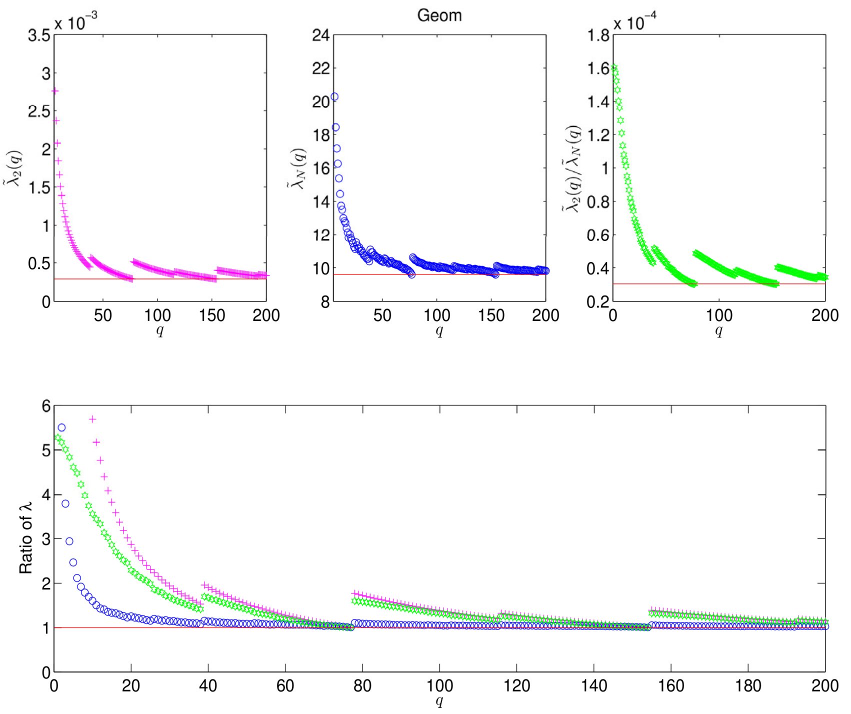
Geom
x 10^-3
$\tilde{\lambda}_2(q)$
$\tilde{\lambda}_N(q)$
x 10^-4
$\tilde{\lambda}_2(q)/\tilde{\lambda}_N(q)$
q
Ratio of λ

(b)

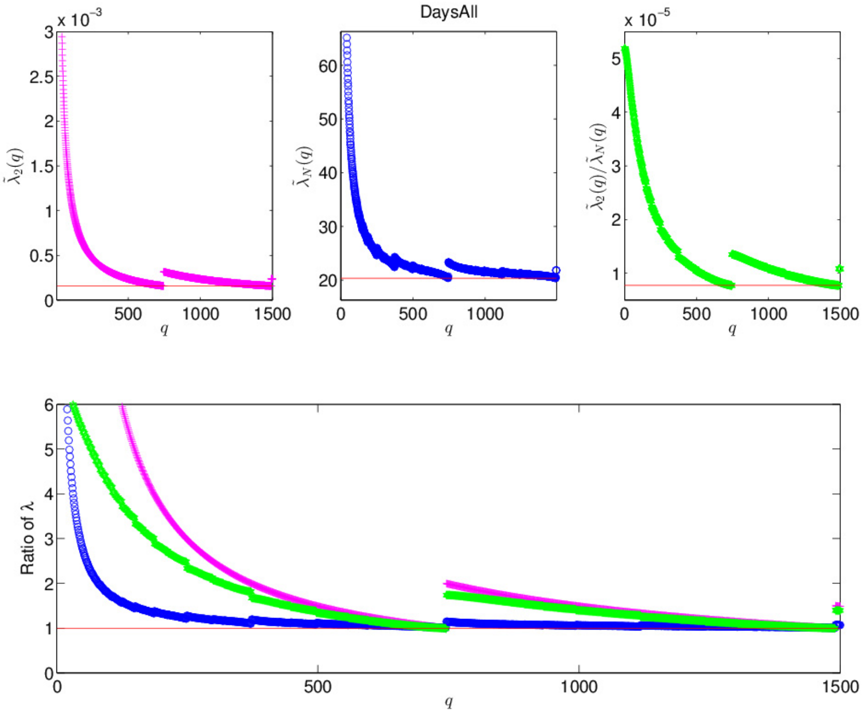
DaysAll
x 10^-3
$\tilde{\lambda}_2(q)$
$\tilde{\lambda}_N(q)$
x 10^-5
$\tilde{\lambda}_2(q)/\tilde{\lambda}_N(q)$
q
Ratio of λ

(c)

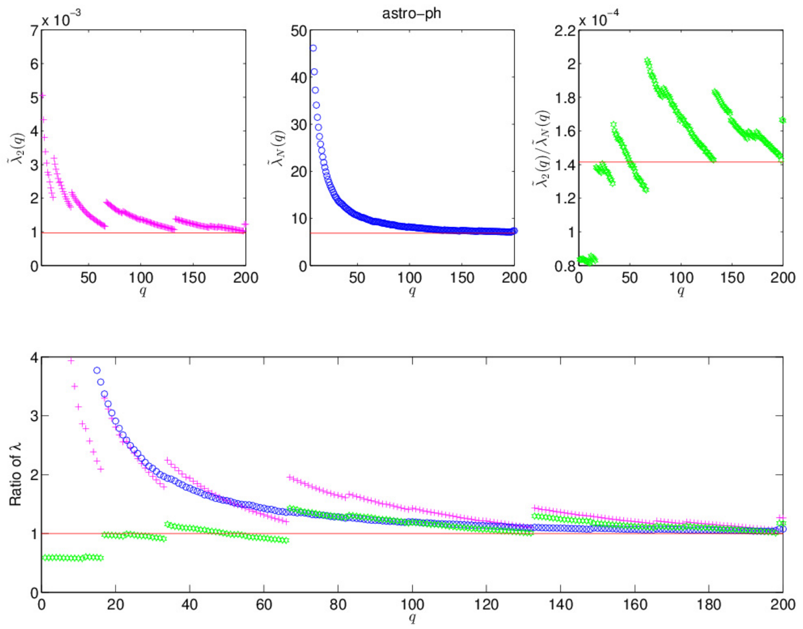

(d)

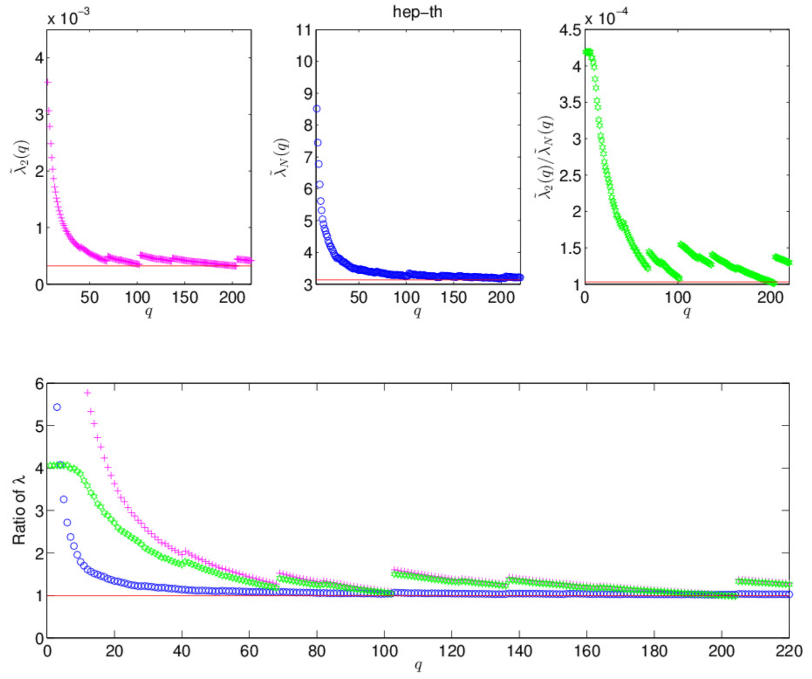

(e)

Figure S1 | Quantization on real-world networks. (a) The Lesmis network (b) The Geom network (c) The DaysAll network (d) The astro-ph network (e) The hep-th network.

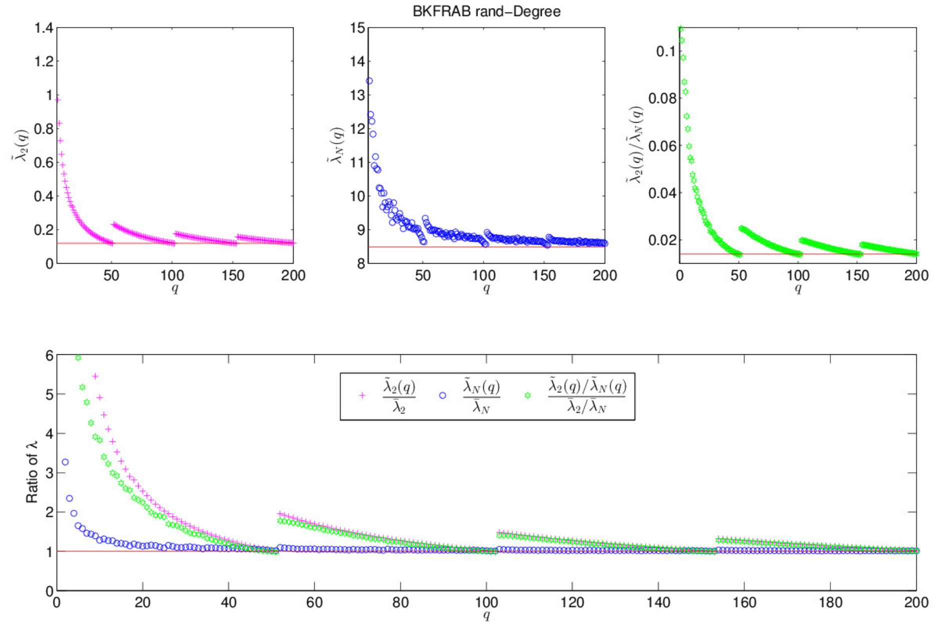
BKFRAB rand−Degree
q
Ratio of λ

(a)

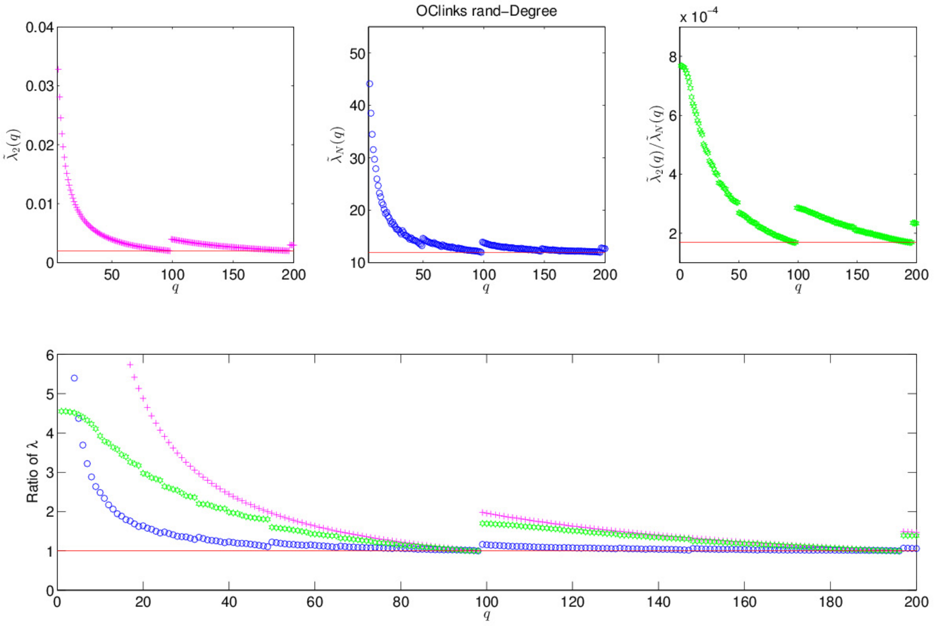
OClinks rand−Degree
q
Ratio of λ

(b)

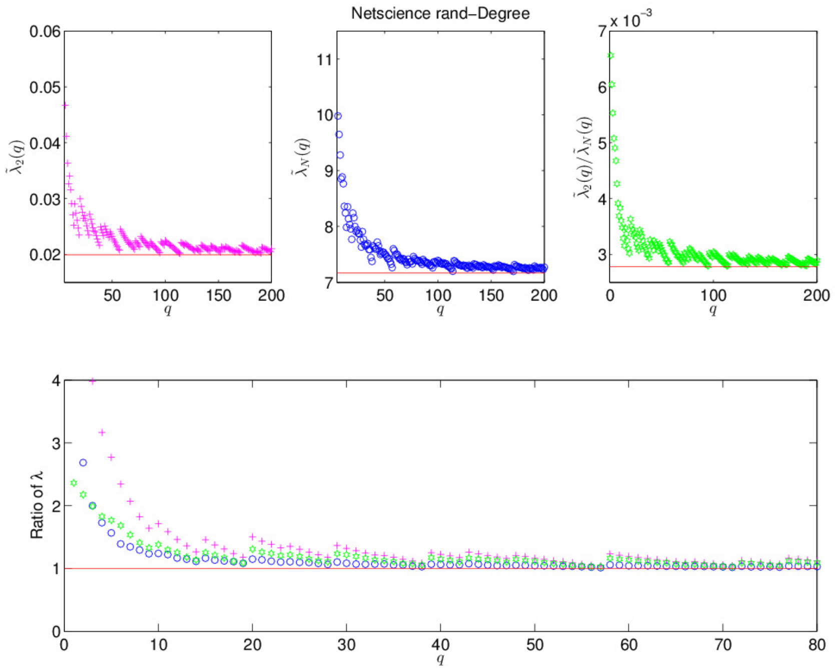

(c)

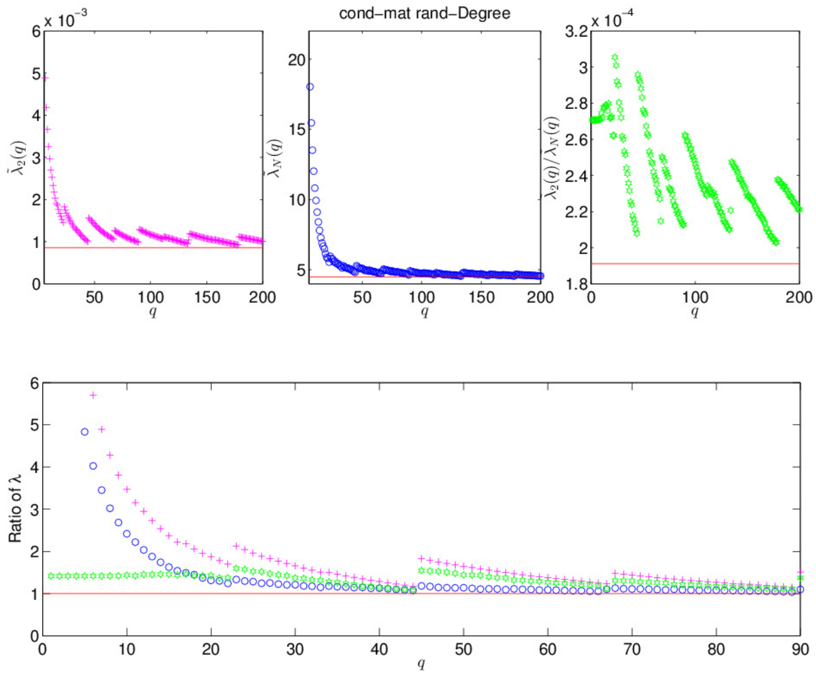

(d)

Figure S2 | Quantization on degree-preserving randomized networks. (a) The BKFRAB network (b) The OClinks network (c) The Netscience network (d) The cond-mat network.

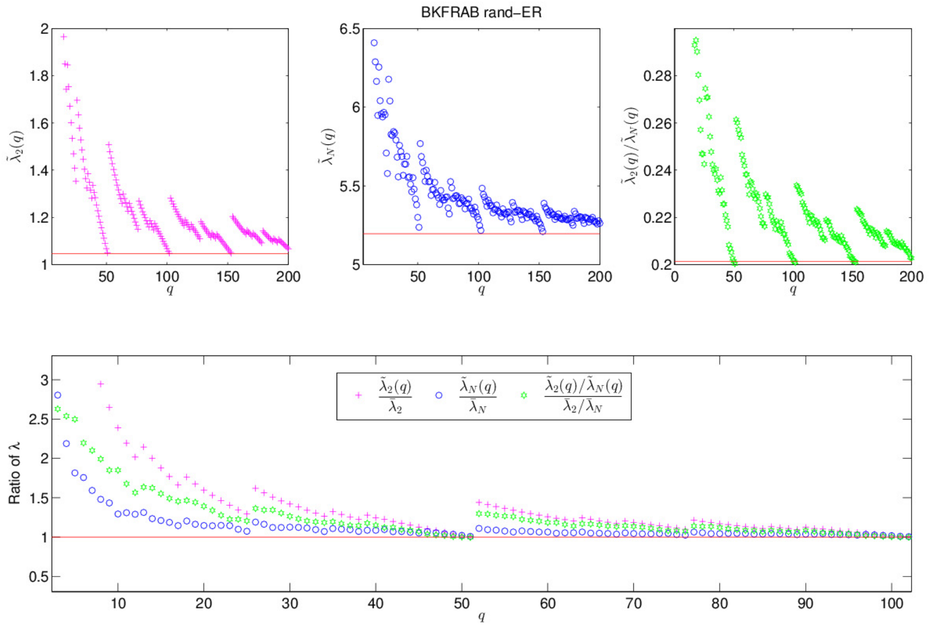

(a)

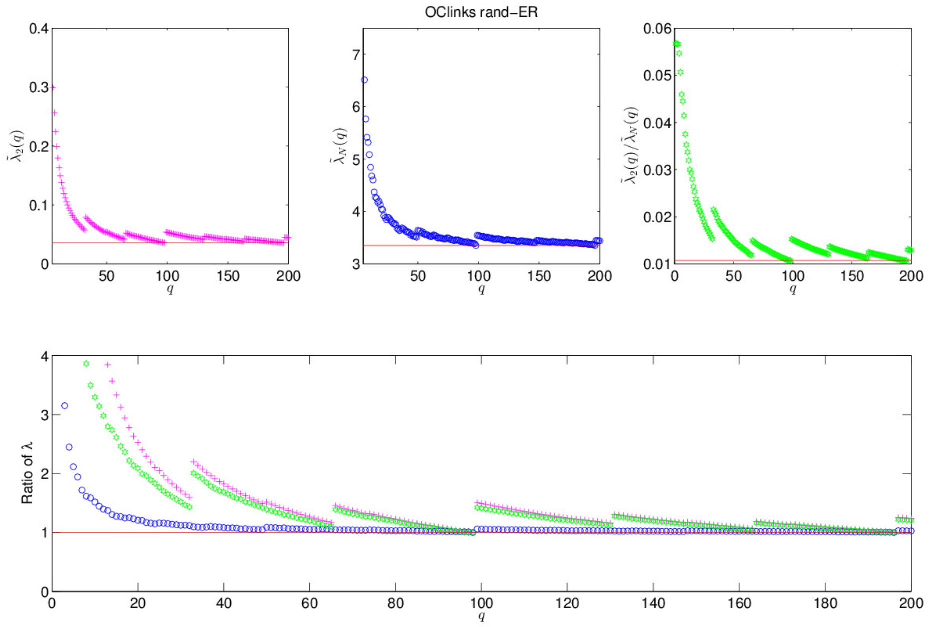

(b)

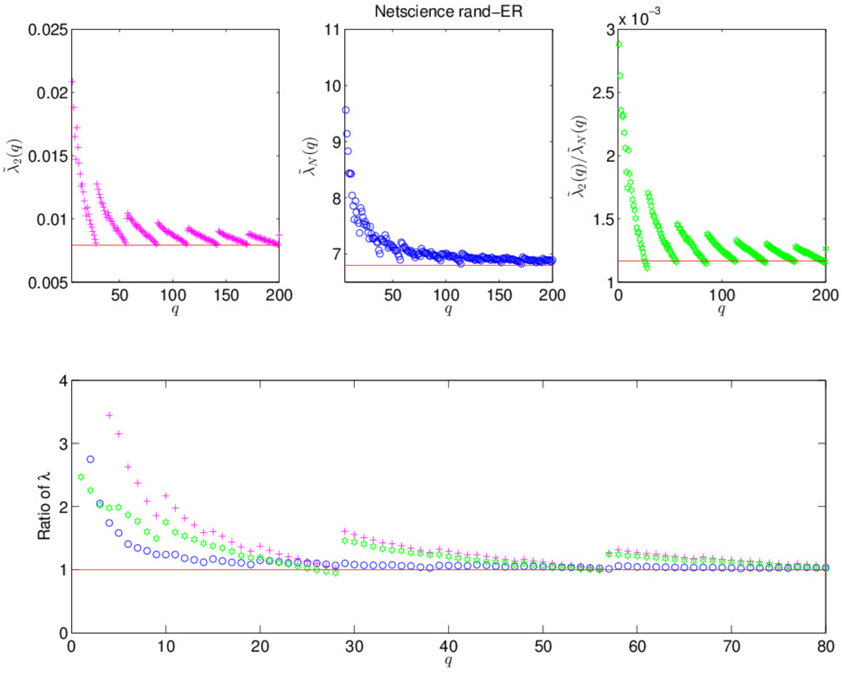

(c)

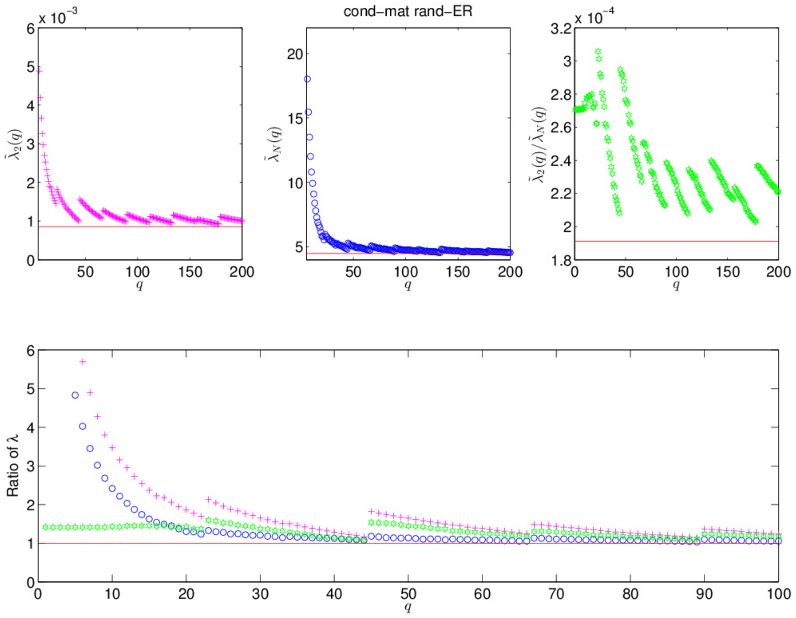

(d)

Figure S3 | Quantization on fully randomized networks. (a) The BKFRAB network (b) The OClinks network (c) The Netscience network (d) The cond-mat network.

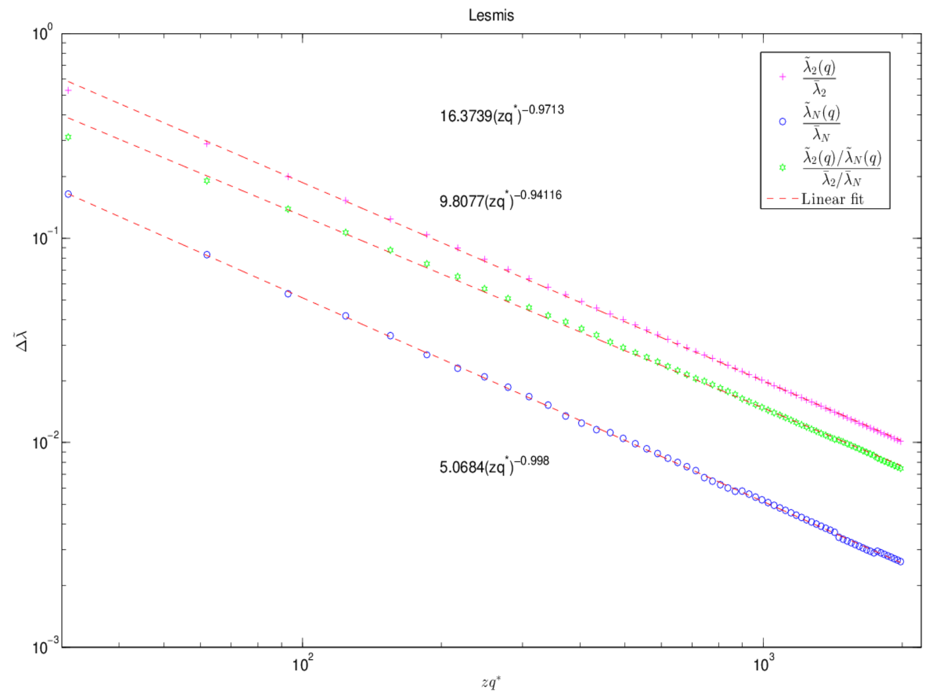
Lesmis
16.3739(zq*)^-0.9713
9.8077(zq*)^-0.94116
5.0684(zq*)^-0.998
Linear fit
zq*

(a)

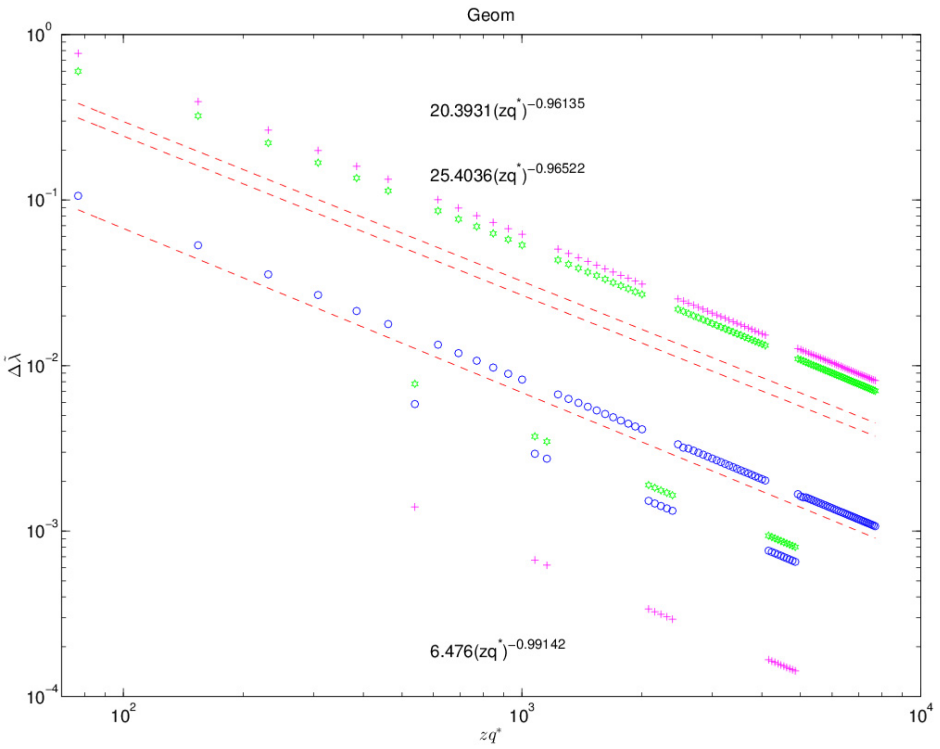
Geom
20.3931(zq*)^-0.96135
25.4036(zq*)^-0.96522
6.476(zq*)^-0.99142
zq*

(b)

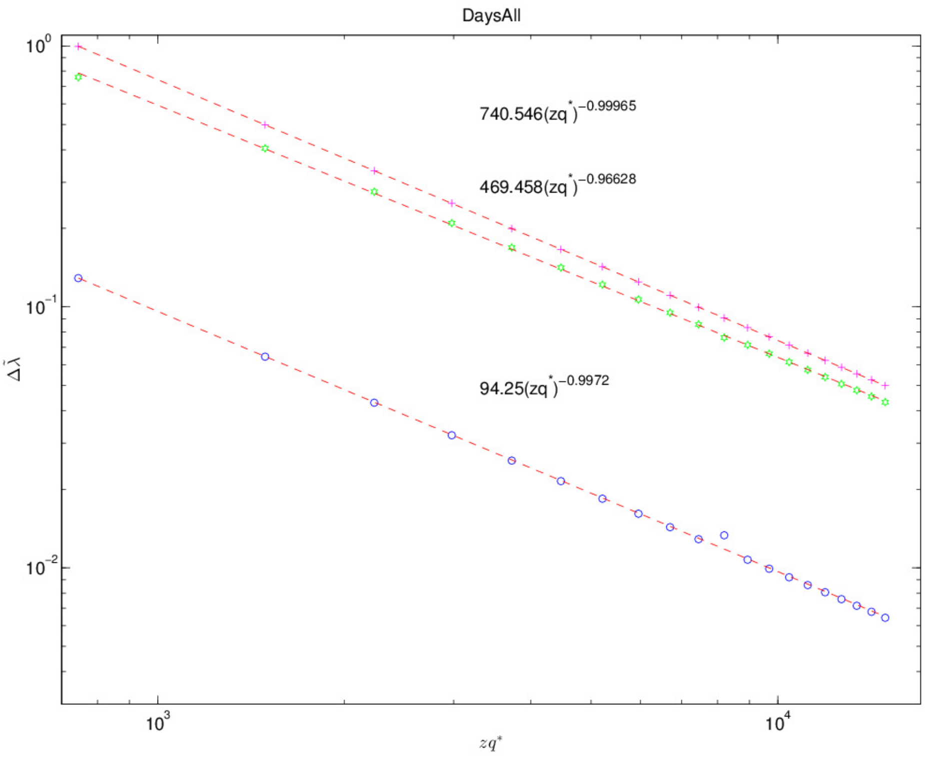
DaysAll
740.546(zq*)^−0.99965
469.458(zq*)^−0.96628
94.25(zq*)^−0.9972
Δλ̃
zq*
10^0
10^−1
10^−2
10^3
10^4

(c)

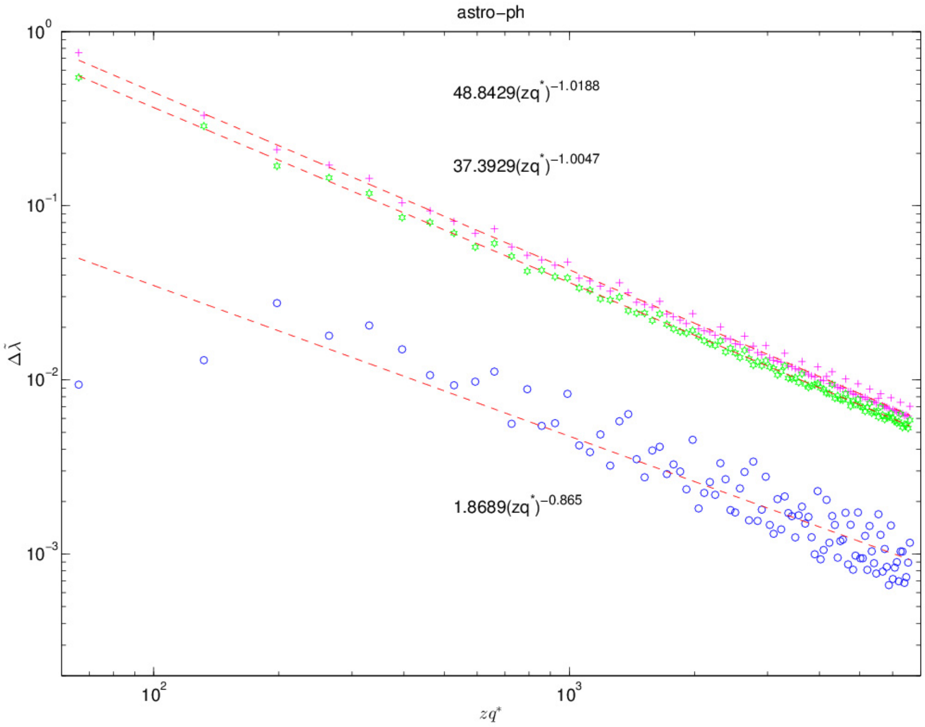
astro−ph
48.8429(zq*)^−1.0188
37.3929(zq*)^−1.0047
1.8689(zq*)^−0.865
Δλ̃
zq*
10^0
10^−1
10^−2
10^−3
10^2
10^3

(d)

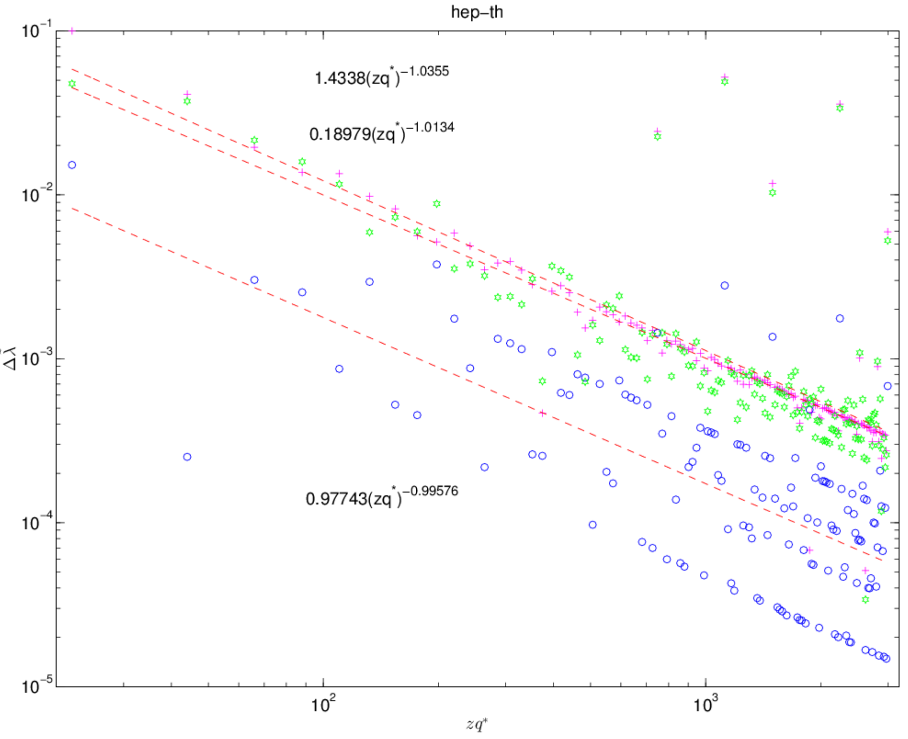

(e)

Figure S4 | Peak values $\Delta\tilde{\lambda}\left(zq^{*}\right)$ v.s. zq^{*}. (a) The Lesmis network (b) The Geom network (c) The DaysAll network (d) The astro-ph network (e) The hep-th network.

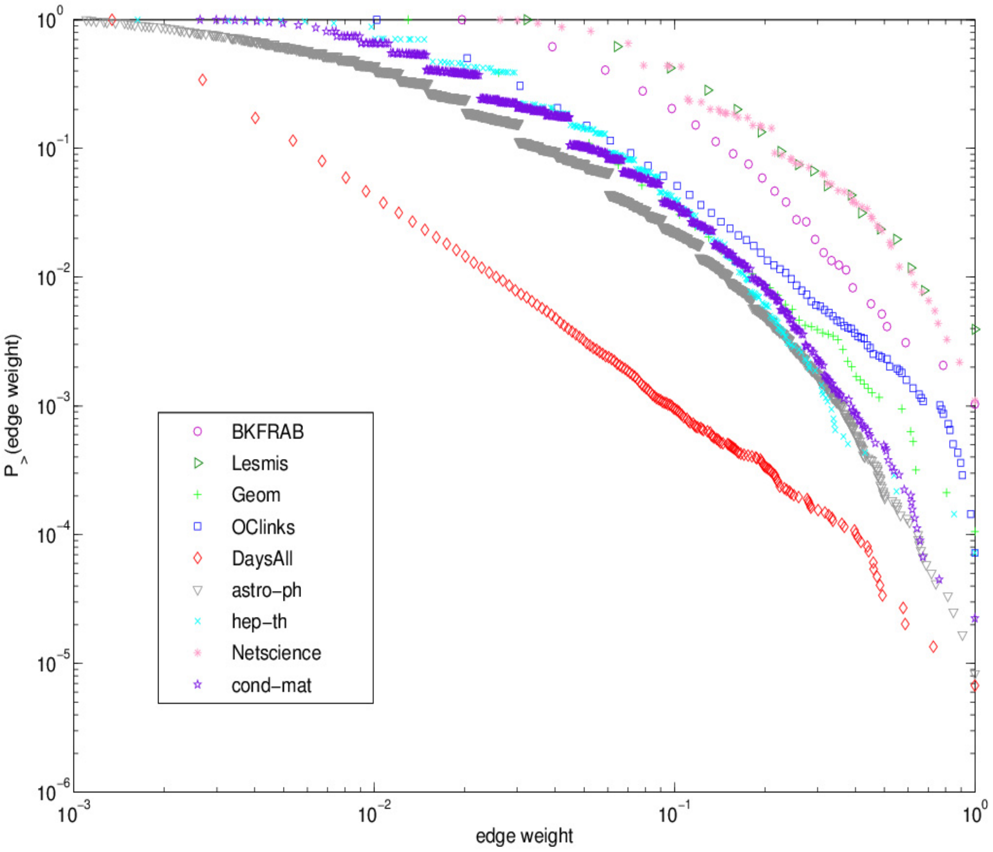

Figure S5 | Cumulative distribution of the normalized edge weights $\bar{w}_{ij}$. The complementary cumulative distribution extracts information from the tail of $P_{\bar{w}_{ij}}$, which enhances the statistical significance of the high edge weight region. If $P_{\bar{w}_{ij}}$ follows the power law $P_{\bar{w}_{ij}} \sim \bar{w}_{ij}{}^{-\alpha}$, then the cumulative distribution scales as $P_{>\bar{w}_{ij}} \sim \bar{w}_{ij}{}^{-\alpha+1}$.

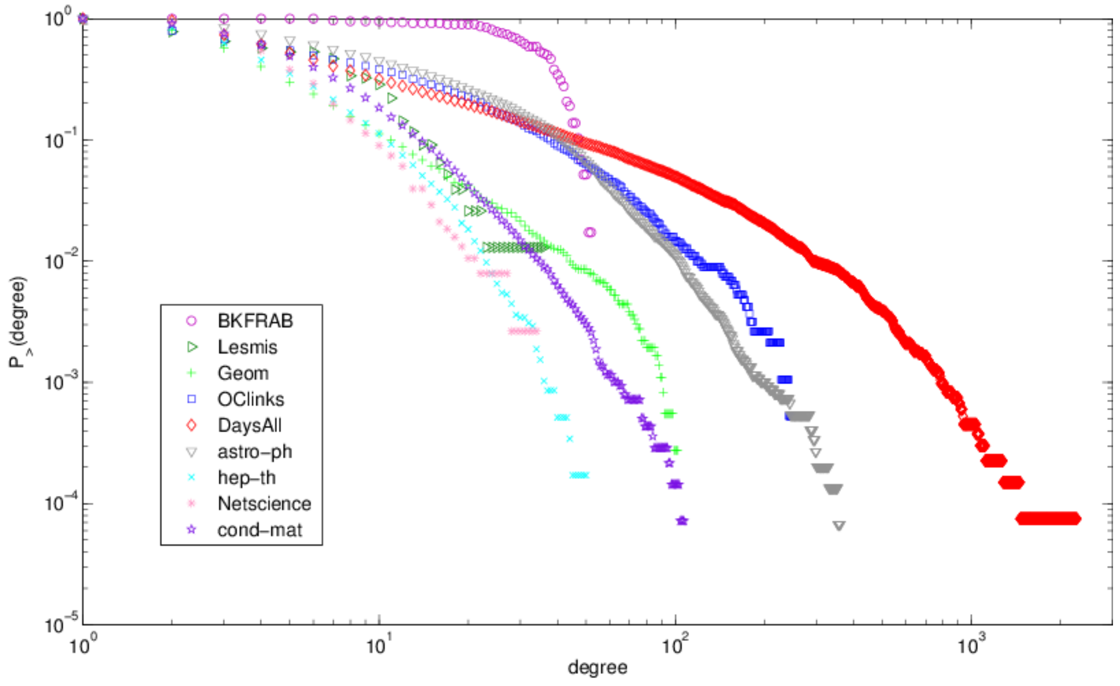

Figure S6 | Cumulative distribution of the degree.

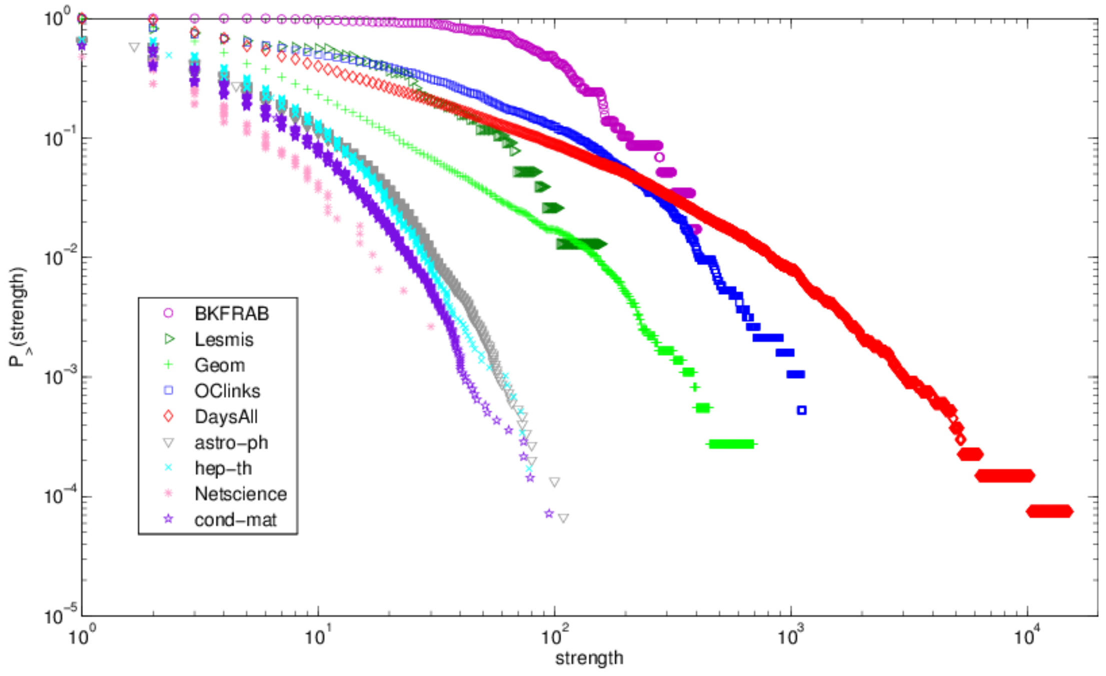

Figure S7 | Cumulative distribution of the weighted degree (strength).

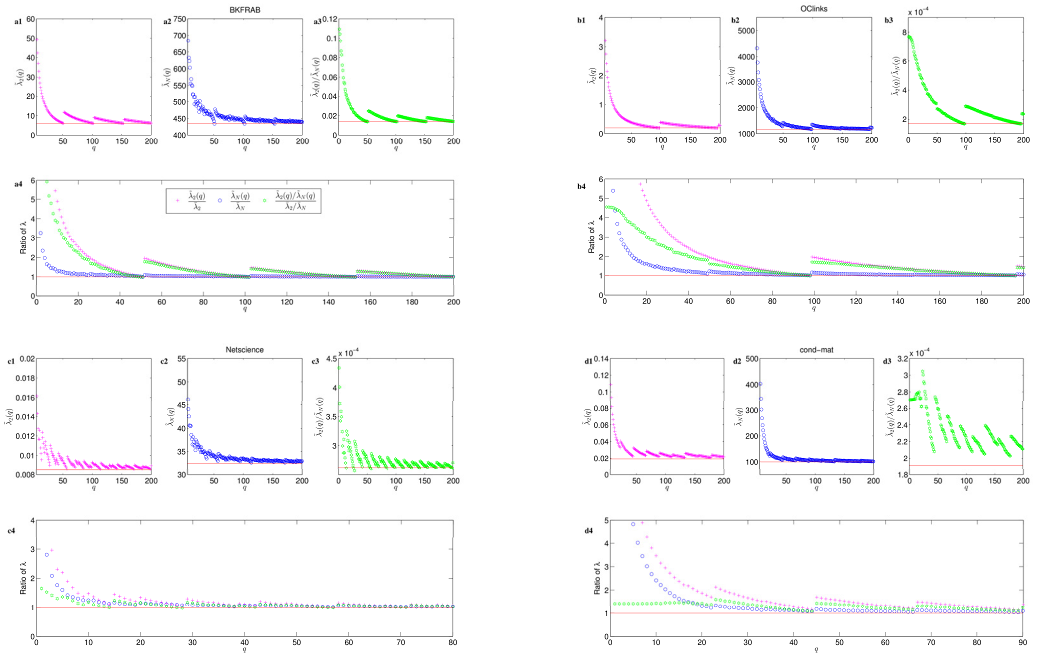

Figure S8 | Quantization on real-world networks without normalization.

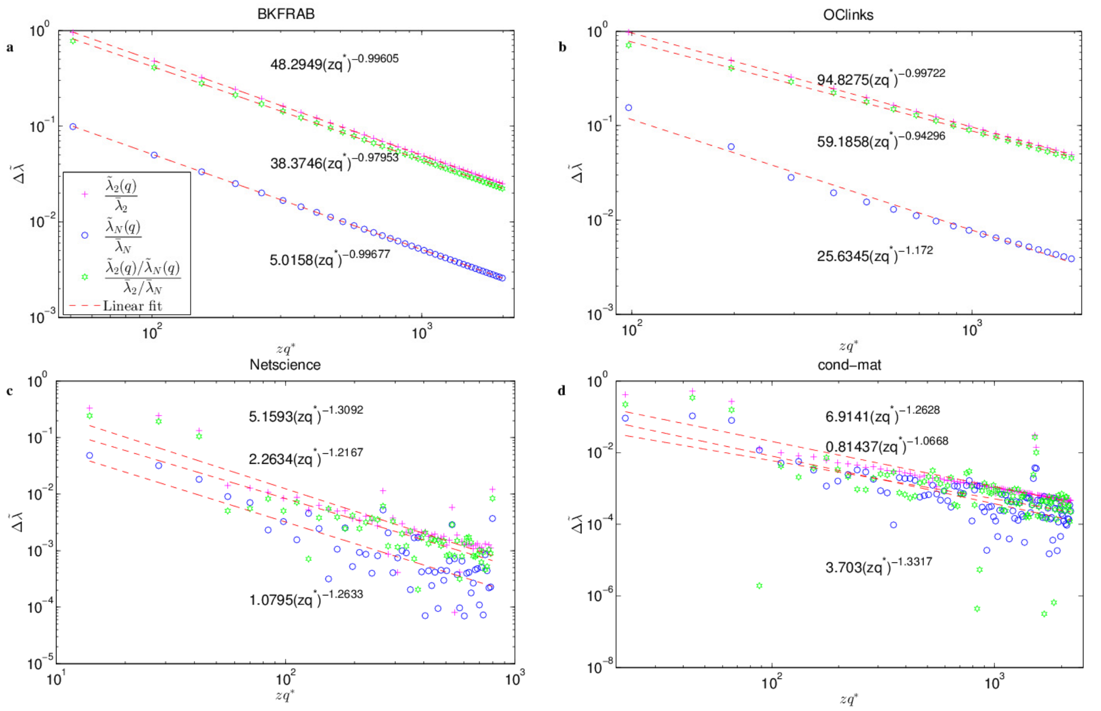

Figure S9 | Peak values $\Delta\tilde{\lambda}\left(zq^{*}\right)$ v.s. zq^{*} of real-world networks without normalization.
